# Local responses in restrictive national policy contexts: welfare provisions for non-removed rejected asylum seekers in Amsterdam, Stockholm and Vienna

**DOI:** 10.1080/01419870.2020.1723671

**Published:** 2020-02-25

**Authors:** Ilker Ataç, Theresa Schütze, Victoria Reitter

**Affiliations:** Department of Political Science, University of Vienna, Vienna, Austria

**Keywords:** Rejected asylum seekers, non-removed persons, municipalities, multi-level governance, sanctuary cities, NGOs

## Abstract

This paper examines municipal responses to restrictive national policies by focussing on welfare provision for non-removed rejected asylum seekers. Using an analytical framework of multi-level governance, we investigate processes of conflict and cooperation and the demarcation of responsibilities between government tiers at the intersection of migration and welfare policy. In an in-depth analysis of the cases of Amsterdam, Vienna and Stockholm, we argue that in order to explain the divergences of public welfare provisions for non-removed rejected asylum seekers, it is necessary to look into their respective legal-institutional framework and formal competences, but also beyond, meaning into the relations of those municipalities with civil society actors and other local governments. We find that, on the one hand, the relationship between local NGOs and the municipality has an influence on the scope of services provided and that, on the other hand, alliance-building between municipalities is crucial for strengthening a political standing.

The involvement of local governments in providing welfare services to irregular migrants exemplifies how cities have become important actors in migration policy; even though their involvement may be distinctly at odds with national policies (Spencer 2017). The inclusive practices of cities, especially towards irregular migrants, against exclusionary national migration policies have been discussed under the label sanctuary cities and solidarity cities, thereby particularly emphasizing the role of non-governmental actors and social movements (Lippert and Rehaag 2013; Squire and Bagelman 2012). Another approach, that of multi-level governance (MLG) explores the importance of the vertical level, i.e. the relationship between national and local tiers, the allocation of competences therein and processes of consensus or conflict. Its strength lies in the thorough consideration of institutional arrangements. We aim to move beyond this perspective by broadening our focus based on insights from the literature on sanctuary cities and integrating the role and contribution of *horizontal* relations such as those with non-governmental actors and those between different local governments into the MLG model.

Based on these analytical considerations, this article examines the responses of three European capitals, Amsterdam, Stockholm and Vienna, to restrictive national policies that attempt to exclude non-removed rejected asylum seekers (NRAS) from welfare benefits. NRAS are persons that still live within the territory after having received a negative response on their asylum request. We consider NRAS to be a subcategory of irregular migrants, since they are subject to the obligation to leave the country and bear no legal right to stay (Ataç and Rosenberger 2019; Triandafyllidou 2010, 6). By local response, we mean on the one hand, the welfare services that are (still or *a fortiori*) offered at the local level despite restrictive national policies and, on the other hand, the political responses of local governments to challenge these policies or even broaden their mandate. Assuming that the competences of municipalities play an essential role, we want to explore municipalities’ responses when national policies restrict NRAS’ entitlements to welfare services. Do they challenge these policies or just act within the scope of their institutional framework? We start with exploring the different courses of action that municipalities choose against the backdrop of differing competences. Through our three cases, we want to show that the horizontal level – the local NGO-landscape as well as networks of municipalities and regions – plays a decisive role in the municipalities’ agency and its limits. We find that insights on the horizontal relations are important for understanding under which conditions local governments may or may not counter national policies, both through service provision and politicization.

Policies towards NRAS and irregular migrants are often ascribed to migration policy and are, therefore, associated with national politics. When national policies restrict access to welfare services for this group as a means of migration control, this can lead to conflicts between the imperative of social policy and the aims of immigration control (Ataç 2019). In contrast to the migration control framework through which national governments may approach welfare policies for NRAS, local governments are likely to come up with a different problem definition and solution, since they are confronted with the consequential problems of denying social support, such as destitution, homelessness, and dysfunctionalities in the public order (Spencer 2017). In this sense, some scholars claim that local migration politics tend to be more “pragmatic” than national ones (e.g. Scholten 2013, 226, 232). However, with few exceptions (see Kos, Maussen, and Doomernik 2015; Spencer 2017; Spencer and Delvino 2019; Delvino 2017), reactions of local governments to restrictive national policies towards irregular migrants or NRAS constitute a widely unexplored field of study (cf. Schiller 2015, 1120).

In the following, we first outline the theoretical foundations, then present our methods and case selection. The main section of this paper starts with a synoptic overview of the three cities’ responses. Subsequently, we unravel the cities’ embedment in multi-level relations to analyse the importance of horizontal relations to other local and regional governments and to civil society actors.

## 1. Theoretical approach

Hepburn and Zapata-Barrero (2014) argue that sub-state levels of government started to steadily gain influence in migration policy, especially in the delivery of welfare services for irregular migrants, a development also referred to as the “local turn”. This mandate is usually related to the municipality’s overall responsibility for providing welfare services. However, even within the same country municipalities may enjoy different leeway in providing services for irregular migrants (Delvino 2017), which indicates that their institutional agency cannot be solely conceived as the deductive product of a national framework. Also, the vertical relation between state and sub-state tiers, which reflects to what extent local governments *claim* that agency (potentially against the central government’s resistance), may take several different forms inside one national framework. This is to say, cities act differently despite a certain focus in academic literature on inclusive cities. The potential of focussing on the local lies in challenging the “assumed monopoly” of the national level in migration policy (Schiller 2015, 1122).

Scholten has developed four ideal types of how this vertical relationship can play out in the multi-level governance: the *centralist* (or *top-down*), the *effective* (cooperative), the *localist*, and finally the *decoupling* mode of governance (2013, 218–221). When applying Scholten’s model to policies concerning irregular migration in Europe, Spencer (2017) finds two major types of outcomes: Either a negotiated solution through institutional arrangements emerges between the local and national tiers. Or, much more commonly, different frames and priorities between the local and national tiers lead to a continued “decoupling”. Some municipalities avoid open conflict by keeping visibility of their actions low, a strategy Guiraudon has identified as “shadow politics” (2004). However, open conflict may arise, when local governments provide access to services despite restrictive national policy.

The literature on sanctuary cities reframes membership at the urban level as an inclusive practice. Two dimensions in this conceptualization provide relevant points of reference for expanding the horizontal dimension of the MLG concept: (a) activities of city governments, e.g. when municipal governments oppose exclusionary national immigration policies, hinder the implementation of these policies and act inclusive towards these groups; (b) non-governmental local actors, e.g. when civil society actors outline inclusionary policies and discourses and reframe a meaning of belonging at the urban scale (Bauder and Gonzalez 2018; Lippert and Rehaag 2013).

Based on Kos, Maussen, and Doomernik (2015) and Delvino (2017), we carve out two important dimensions of local responses to welfare-restricting national policies: political responses and alternative local service provision. The former grasps actions to build up social and political pressure by mobilizing public and political actors. It takes on a signalling function by speaking out against the ineffectiveness and moral wrongness of national policies in alliance with advocacy organizations and other municipalities. This dimension of municipal responses is connected to relations of municipalities with other municipalities: horizontal processes connecting multiple local governments (Filomeno 2017; Spencer 2017). Local policy makers increasingly focus on alliance-building when it comes to intractable issues such as services for irregular migrants, which require the formation of relational networks and strategic partnerships among different actors (Ambrosini and Boccagni 2015).

As the second dimension of municipal responses, we denote the development of alternative and/or complementary practices of service provision. Ambrosini and Boccagni (2015) emphasize the NGOs’ role as intermediaries between local authorities and migrants in the provision of services. With the importance of local actors growing, city governments started to collaborate with civil society actors to implement social programmes (Nicholls 2006). Municipalities prefer to outsource services to NGOs to expand their outreach and impact, as well as to delegate the risk of breaching national rules (Delvino 2017, 36). Considering this involvement, NGOs are contributors to local welfare arrangements and, as such, part of the governance structures. The incorporation of civil society organizations into governance processes can occur in two forms: either in tight collaboration with municipalities, e.g. as implementation partners, or as independent entities. In the latter, civil society organizations act as autonomous organizations through offering services but also by forming advocacy coalitions and participating in public policy arenas. With this theoretical framing we join a recent literary strand, which highlights the importance of horizontal relations in multi-level governance constellations (Campomori and Caponio 2017).

Informed by this theoretical debate, which delivers insights on the role of horizontal relations and their impact on the agency of the cities, we analyse how the relations of three selected cities with NGOs and among each other influence their responses towards NRAS’ access to welfare services in restrictive national policy contexts. The debate on different types of vertical relationships in an MLG setting helps us to locate the role and the scope of action of local governments in each national setting. The different responses of the cities, with respect to their diverging competences, and their embeddedness in different MLG models are our starting point. Then, following scholarly discussions of the “local turn”, we discuss how local government networks and NGOs, whether as independent actors or commissioned by the city governments, are becoming part of the MLG, and, hence, of the local response towards national policies. Following the literature on sanctuary and solidarity cities by including the relation to civil society actors in the analysis of the cities’ responses also helps to go beyond a narrow policy-oriented understanding of migration governance. At the same time, we employ the MLG-lense for its strength to enable more thorough knowledge on the interaction between political and societal institutions and their effects on political decisions.

## 2. Data and methods

This paper bases on case studies informed by a comparative perspective. The data for this paper were compiled within a research project investigating access to welfare services for NRAS in Austria, the Netherlands and Sweden. The data were collected through a range of semi-structured qualitative expert interviews with national and municipal government officials in charge of making decisions on, or with expertise in, access to welfare benefits for NRAS. In addition, interviews were conducted with academics and lawyers with insight into the relationships between the local and national level as well as between local governments and civil society, and other stakeholders such as NGO workers. Overall, 73 interviews were conducted between June 2016 and July 2017, which provide diverse perspectives on the delivery of services, the relation and interaction between the national and local tiers, as well as between NGOs and cities. The interviews were transcribed, coded with atlas.ti, and analysed using the content analysis method.

In addition to the interviews, our body of empirical data also includes policy documents such as legal commentaries, legislative texts, laws and parliamentary documents, as well as decisions by international courts and statements by human rights institutions. These documents provided context and were used to further explicate important aspects that came up in the interviews. We enriched the data by including a review of secondary literature. Based on our analytical focus on MLG-relations, we structured the data in analytical templates for each city with information on formal responsibilities, detailed timelines of actions taken by municipalities in the context of the implementation of restrictive national policies, and information on the motivations and arguments of the municipalities. Based on our data structured in the templates, we identified the relations to civil society actors and other municipalities as recurring and crucial aspects shaping the cities’ actions. With this refined focus, for all three cities, we further mapped out civil society activities (services as well as mobilizations) for NRAS, the relations between these actors and the municipality, and the city’s relation with other cities. Comparative aspects in these horizontal relations were, regarding the NGOs, the degree of institutionalization of civil society activities and their incorporation in municipal welfare programmes for NRAS, and, in regard to cross-municipal cooperation, the degree of collective organization as well as the purpose of networking.

## 3. Non-removed rejected asylum seekers in Sweden, Austria and the Netherlands

Although NRAS can be considered as a subcategory of irregular migrants, their situation is certainly specific. On the one hand, NRAS usually do not receive a legal residence permit in an EU country (Lutz 2018) and the EU Return Directive (2008/115/EC) does not require states to issue such permits. While Article 14 of the Return Directive advocates some minimum rights for NRAS, including family unity, emergency health care, respect for situations of vulnerability, education and documentation, it does not cover other essential rights such as accommodation. Consequently, the EU Member States maintain plenty of room to manoeuvre (Schoukens and Buttiens 2017). As NRAS are subject to welfare benefits including housing during their asylum procedure, following a negative asylum decision many face homelessness. In most European countries, NRAS have no legal access to the labour market (Lutz 2018), which contributes to the risk to end up living on the street.

Our selection of Amsterdam, Stockholm and Vienna as cases for this study is primarily based on their situation in a national context that has recently been the site of restrictive developments related to NRAS at the national government level, as evidenced in jurisdiction, policy and political debate. In the Netherlands, exclusionary welfare policies against irregular migrants in general date back to the Linking Act of 1998. However, in recent years the topic has resurfaced when the Dutch government explicitly excluded NRAS from shelters and tried to prevent municipalities from providing shelter to this group. The Swedish government eliminated access to social benefits for rejected asylum seekers in 2016, through a change of the Swedish Asylum Seekers Reception Act (LMA). According to the Swedish Migration Agency (SMA), some 4,000 NRAS have lost social support since the introduction of the new regulation (Interview 10). Austria is one of the few countries in Europe in which NRAS have access to state-organized accommodation. The national government has since 2016 demanded the exclusion of NRAS from welfare benefits, resulting in a change of the distinctly federal Basic Welfare Support Act in 2017 and leading to conflicts between, inter alia, Vienna and the national government.

Because of national governments’ restrictive stance towards NRAS, local governments experience increased pressure to avoid a provision vacuum and negative social ramification. We chose to focus on how this plays out in the cities of Amsterdam, Stockholm and Vienna, because – as for all migrants with irregular status – larger cities tend to offer more opportunities and therefore act as magnets. Each of these cities represents the most populous in their national context and all share the experience of an ongoing presence of irregular migrants as well as NRAS.

While the selected municipalities share some common features that substantiate a presumed need for action, their institutional agency is differently shaped since they bear differing official responsibility for dealing with this group. As Vienna is both a federal province and Austria’s largest cities, it has strong implementation power in the field of integration and social policies, including responsibility for the effective provision of welfare services for NRAS. In the Netherlands and Sweden, the national level is solely responsible for the provision of housing and other welfare services for rejected asylum seekers. Accordingly, the municipalities of Stockholm and Amsterdam bear no formal responsibility for NRAS regarding welfare services relevant to the scope of our research.

## 4. Municipal responses to restrictive national policies

In the following, we outline the municipalities’ responses in their two dimensions: (a) offering services to individuals and (b) their political responses. This includes looking at the overlapping mandates between the national and sub-state tiers. The overall responses illustrate, how the municipalities either exhaust, overstep or act within their legally determined scope of action.

### 4.1. Vienna: fiercely defending an effective MLG

The Austrian Basic Welfare Agreement is an institutionally anchored arrangement. It defines a division of labour between the national and sub-state tiers, making NRAS an explicit target group of welfare support. The provinces are responsible for its implementation and each province has its own law based on the agreement, which gives the provincial governments room to adapt their own legal specification.

Among the Austrian provinces, the highest number of NRAS resides in Vienna. In the past decade, Vienna was one of the few regions that fulfils and exceeds the nationally organized quota system that distributes asylum seekers per province. As other provinces were more restrictive, many of those affected moved to Vienna. In these cases, the welfare department of the city of Vienna (FSW) used its discretionary power to grant services: they extended services in cases of hardship, considering especially medical cases and local family ties, and included NRAS who are registered in the city but are not actually covered by its mandate (Interview 18, 19; Ataç 2019). A managerial employee of the implementing NGO stated about the political intention behind this action that “(it is) quite clearly to avoid homelessness, to not promote delinquency” (Interview 18).

Vienna’s generous implementation practice sparks disagreements with the interior ministry. One such disagreement revolves around the issue of cooperation with return, which presents a condition for being granted access to the welfare support system. In case of alleged violation of the cooperation duty, the Federal Agency of Migration (BFA) requests that provinces dismiss the individual from the support system (Rosenberger and Koppes 2018). The FSW has kept these persons in the system nevertheless by arguing that the factual presence of NRAS makes them subject to the welfare agreement (Interview 21). However, when the municipality of Vienna grants these “extra” services, it does so in the form of shadow politics (Spencer 2014; Guiraudon 2004), meaning that it does not actively enhance the visibility of services and debates.

Having said this, Vienna does become publicly outspoken, when the guarantees of the welfare agreement seem at risk. After the summer of 2015, when asylum applications were exceptionally high, the federal government argued for cutting access to welfare services for NRAS, and Vienna became pro-actively involved in politicizing the issue: Vienna’s speaker for welfare issues was one of the strongest voices to challenge the welfare cuts by proclaiming that a limitation of access to welfare support would put thousands of NRAS on the street. Likewise, in parliamentary debates, the mayor of Vienna employs what Spencer and Delvino term an inclusive security frame (cf. Spencer and Delvino 2019). He explains, that the city must offer services “because our opinion is we cannot leave people on the street (as homeless)” (parliamentary debate, 27 January 2012).

To sum up, Vienna exhausts the limits of its implementation power and sometimes tiptoes around its legal boundaries. Gatekeepers describe the practice in Vienna as a pragmatic approach. As one key worker at the responsible organization states: “As long as (NRAS) are there, (they) get basic care, have very pragmatic access. This means that most of those (NRAS) who are in Vienna do not lose access to basic care at all” (Interview 19). In so doing, the city facilitates a comparatively high level of access to social services for non-removed persons. Politically, it is more re-active than proactive, but steadily defends its policies when conflicts arise. We therefore see a two-sided approach of shadow politics (Guiraudon 2004) when it comes to service provision for cases of hardship, combined with entrenchment in the legal framework.

### 4.2. Amsterdam: decoupling and counter-politics

In the Netherlands, the issue of welfare services for NRAS is marked by a history of conflictual processes that shape the relationship between the Dutch national government and the municipality of Amsterdam. Through the instalment of the Linking Act in 1998, the Dutch national government had established the legal grounds to cut access to public welfare provisions for irregular migrants including NRAS (Van Meeteren 2014, 66; Van der Leun 2003). Based on the Linking Act, the Dutch government has repeatedly tried to prevent municipalities from offering welfare services and particularly accommodation to NRAS (Oomen and Baumgärtel 2018; Kos, Maussen, and Doomernik 2015).

Before 2014, only exceptional cases considered extremely vulnerable had access to the shelter programme in Amsterdam and most NRAS were left to the streets (Interview 28). The contradicting approaches of Amsterdam and the national government have resulted in open non-compliance between both tiers. In 2014, a ruling of the European Court of Social Rights (2014) supported the municipalities’ position: The ECSR decided that the central government’s prohibition of social services to homeless irregular migrants presented a violation of the Dutch obligations under the European Social Charter (Oomen and Baumgärtel 2018, 617). This opened a window for Amsterdam and other Dutch municipalities to install or extend temporary accommodation facilities, which became known as the “Bed-Bad-Brood” or “BBB-Scheme” (Schapendonk 2016, 118).

In 2015, the national government, in turn, attempted to restrict this scheme; it planned to close all municipal shelter facilities except for five facilities, which should only be accessed under the condition of cooperation with return (Oomen and Baumgärtel 2018, 618; Schapendonk 2016, 122). Municipalities, however, protested this so-called BBB-compromise, which in their view was a paper solution that did not fit local realities and was even deemed “insane” (“krankzinnig”) (*de Volkskrant*, 23 April 2017). After several rounds of negotiations, an agreement could not be reached between local and national government (Letter to Parliament, 29 November 2016). In 2016, the Dutch Administrative High Court supported the stance of the national government by ruling that municipalities had neither the duty nor the power to provide shelter to undocumented migrants and defined the shelter provided by the city of Amsterdam as an “extra-legal” policy (Oomen and Baumgärtel 2018, 619; Delvino 2017, 14f). The State Secretary of Security and Justice reacted by retracting financial support of BBB facilities. Still, Amsterdam, like most municipalities, has continued to operate shelter facilities out of its own budgetary means (Interview 24).

Spencer classifies the Dutch situation, where sub-state tiers provide access to a service despite resistance by the national government, as overt conflict (Spencer 2017, 11). Speaking with Scholten’s ideal-types (2013), Amsterdam provoked decoupling by contesting the top-down MLG setting through offering the Bed-Bad-Brood scheme. The national government has argued that the denial of shelter for NRAS was a necessary policy for disincentivizing irregular stay and rendering return policy more effective (Leerkes 2016, 144), thus making it a matter of migration policy. Amsterdam has challenged this approach by emphasizing its mandate to guarantee public order and safety, as well as public health (Interview 23; Kos, Maussen, and Doomernik 2015, 9f), and that the provision of services presents a necessary step to achieve these goals. Accordingly, the responsible policy officer for Amsterdam stated in an interview “we do this … on the basis of public order and safety … and based on the humanitarian safety net, that government does not want people to sleep on the streets (…)” (Interview 26).

In sum, Amsterdam offers basic reception services as part of its official municipal policy. The municipal government keeps being politically “loud” in the ongoing nationwide conflict concerning the responsibility to provide such reception, which is rejected by the national government. We, therefore, characterize Amsterdam’s approach as discursive and policy-based counter-politics.

### 4.3. Stockholm: a non-issue in a top-down setting

Since 2002, the Swedish government had continuously extended social rights for migrants living in irregular situations (Lundberg and Spång 2017, 52). This development peaked in 2013, when statutory changes were passed increasing rights of irregular migrants (and hence to NRAS) to health care and education for minors (EMN 2016, 15). However, in 2016, as a reaction to the sharp increase of asylum applications in 2015, the national government eliminated access to welfare benefits for NRAS (LMA 1994, §11 and §11a). This led to uncertainties among municipalities regarding their responsibility according to the Social Service Act, specifically whether this extends to NRAS requiring emergency care (Interview 2).

Only one and a half years after the LMA-amendment (law on the reception of asylum seekers) was passed, Stockholm issued new guidelines to its social service offices: First, to widen access to services for children independent of their and their parents’ legal status and, second, to underline the mere possibility of social service offices to assist undocumented migrants and NRAS in emergency situations, in accordance with §2 of the Social Services Act 2001 (Utlåtande 2017, 222). The guidelines’ practical relevance is limited, since families with children are exempted from the restrictive turn at the national level either way. Emergency support for NRAS is only granted in very few cases and may likely consist merely of shelter for single nights or food for the day (Interview 9, 11, 12). Local welfare officers argued that they do not want to counteract the national regulations, even though they have relatively high decision-making power due to the principle of local self-government. Likewise, a bureaucrat in the Stockholm social policy administration stressed that “it's also a problem. […] We are an authority and they are an authority and we decide in different directions” (Interview 5, see also Interview 2, 8, 14).

Access to social services for NRAS has so far been successfully framed as a migration policy problem by the national government. The city of Stockholm issued no comment on the amendment of the LMA (mail correspondence, 21 February 2018). It remained silent and has not politically opposed the change of the LMA law. This arrangement most closely reflects Scholten’s ideal-type of a vertical top-down relationship. The important difference to Amsterdam is that Stockholm accepted the top-down governance and implicitly accepted the national migration policy labelling by making it to a non-issue. It provides only sporadic and fragmented patchwork-like support of individual cases of hardship of NRAS through its district welfare offices.

To summarize, we find three “types” of responses in the cities. The following chart provides a synoptic overview these responses and their two-dimensional composition (Table 1) .

**Table 1.** Synoptic overview of cities' responses.

	Vienna	Amsterdam	Stockholm
Mode of multi-level governance	cooperative	decoupling	centralist
Overall “type” of response	entrenchment in MLG-setting and shadow politics	Counter-politics	Non-issue
Local service provision	Comprehensive service system, including health care, accommodation (collective/private), pocket money	Minimal reception as part of official municipal policy (scheme)	Fragmented and sporadic support of individual cases of hardship
Political response	Firm political response, when service arrangement is attacked	Open non-compliance with national policies	No positioning against national policies

## 5. Analysis: the importance of relations in shaping the municipalities’ responses

The legal institutional setting and the architecture of the MLG define the scope of action of the municipalities in all cities and shape access to welfare services for NRAS. The differences between Amsterdam and Vienna are evident in this respect. Although both cities strongly oppose the national policies in this matter, they enact this “opposition” differently: Since Vienna holds strong implementation power granted by the Basic Welfare Agreement, its inclusive approach is covered by its discretionary power and takes place in the institutional setting of the national MLG in form of shadow politics. In Amsterdam, by contrast, different frames and priorities between the local and national tiers lead to decoupling. The MLG is constructed in a centralist top-down way where the national government solely takes the authority and responsibility for the decision of access to welfare services for NRAS. Providing reception in the scope of the BBB-scheme in Amsterdam is the outcome of an open conflict, transgresses formal competences, and remains contested at the national level.

There are strong differences between the responses of Amsterdam and Stockholm, although in both cases, national agencies claim sole responsibility for organizing welfare access to NRAS and both cities bear no formal responsibility for NRAS in the context of the existing MLG. While the city of Amsterdam provides access to services against the opposition from the national government, its official scope for action derives from the mandate of ensuring social order. Likewise, cities in Sweden hold a general mandate for providing social services to all persons residing in the city under the Social Services Act (Interview 3; Cuadra 2015, 308). However, the municipality of Stockholm has refrained from developing a scheme for service provision for NRAS. Rather, it provides only fragmented services, which are at times granted arbitrarily.

Concludingly, cities act – independent of their position in the MLG – more rebelliously or more adjustingly. Hence, we must look beyond the legal duties and the responsibilities to explain the cities’ agency and their actions. In the following section we argue, that the ties between local governments and civil society organizations shape the grounds for service provision and the ties between and across multiple local governments are relevant for shaping municipalities’ politicization of the issue.

### 5.1. Civil society organizations: backbone and intermediary

Starting with the relevance of civil society actors, we argue that the incorporation of NGOs into municipal service schemes creates opportunities for the cities. Both Amsterdam and Vienna outsource service provision to NGOs that existed already prior to the development of their schemes. When the city of Amsterdam started to offer shelter services to NRAS in 2014, it established a cooperation with an NGO, which implemented the Bed-Bath-Bread scheme on its behalf. In Vienna, services are mostly organized through an NGO, which manages the central distribution of the basic welfare support to clients on behalf of the Vienna Welfare Agency (FSW) and runs some of the housing facilities. From the perspective of the city, this close cooperation enables them to fall back on an existing support infrastructure for effective policy implementation and helped to expand their outreach to clients (Interview 16, 17, 25, 27). The city of Vienna profits from this cooperation since it enables the city to implement access to welfare services for NRAS through a form of shadow politics – services are offered without raising public awareness about it.

From the perspective of the incorporated NGOs, their influence on the municipalities’ decisions exists on a more informal level and they can use their position to offer services to persons that otherwise fall through the net. For instance, in Vienna counsellor function as gatekeepers when they push cases of hardship to influence administrative decisions: “Well, there's no legal claim to it, (…) it's always a case-by-case decision, it's a point of argument” (Interview 15). In general, they were emphasizing good relations with the welfare agency of the city (Interview 18, 19). As one NGO-worker put it: “One can argue with them” (Interview 17). Counsellors found themselves with a double mandate of being both service providers for the municipality and representatives of clients’ interests. To fulfil this dual role in their daily routine, the NGO actively transgresses its position as provider for municipal services and fills service gaps: it opened a second service point for clients, who are denied welfare services by the city, such as undocumented and non-removed persons. It finances the service point through donations and uses the knowledge and infrastructure acquired by giving services on behalf of the municipality (Interview 15, 18).

When civil society organizations demand an improvement of welfare services for non-citizens and put pressure on the state in the form of regular protests and claims-making, their power of realizing their goals vis-à-vis local governments increases (de Graauw 2015). When the Dutch Linking Act of 1998 was introduced, excluding irregular migrants from public services, the number of NGOs supporting this group grew. In 2011, the number of NGOs offering shelter to irregular migrants was estimated at approximately 40 active NGOs (Van der Leun and Beuter 2015). Moreover, some NGOs also became active in litigations and took the case successfully to the European Committee of Social Rights when NRAS and their families were evicted from state accommodation centres (PICUM 2014, 16). This leverage effect of NGOs explains what makes decoupling in Amsterdam possible, which moves the city towards a more inclusive policy: On the one hand, NGOs conduct pioneer activities for social rights advocacy and organize campaigns; on the other hand, NGOs have been giving services to NRAS before the municipality started to provide services for this group.

Regarding the relations between municipal authorities and NGOs, the situation in Stockholm differs significantly from Amsterdam and Vienna. In Stockholm, the contribution of NGOs and the churches’ work lies outside of a concerted governance setting. Even though some NGOs and the church of Sweden receive national and municipal funding contributions for their general operations, they neither work on behalf of the municipality nor are NRAS considered a specific target group of that funding (Interview 6). When the new regulation came into effect on 1 June 2016, several NGOs reorganized their operations due to the rise in demand for acute support (Interview 4, 7, 9; Swedish Red Cross 2016). NGOs have also reacted more critically than the municipality of Stockholm to the LMA-amendment pointing to effects like destitution and homelessness (Interview 13). One team leader in an NGO explained: “When we knew that the law would change, we realized that […] this is going to be a big problem […] All the NGOs said that […] there will be a lot more undocumented people out on the streets and they will first come to us” (Interview 9). Besides the initiatives of NGOs, the new national restrictive regulations triggered the emergence of networks on social media platforms through which voluntary private persons and groups are reached and mobilized (Interview 1, 11).

While in all three cities NGOs provided services for NRAS outside of official municipal policy (Interview 6, 20, 28), in Stockholm they are only loosely or not at all incorporated in the service provision of municipalities. Nevertheless, independent civil society initiatives and churches play an important role in compensating for the municipality’s lack of coherent action as “backyard activities” and have likely dampened the local effects of the federal law amendment. However, as with the local social service offices in districts and municipalities, the access to and the quality of services differs widely as it eventually “depends on who opens the door” (Interview 6). In this paper, we discuss local public services and connected humanitarian civil activities that can be labelled pro-irregular-migrants. However, those services must not be romanticized: They are no more than ensuring NRAS’ bare survival, and do not tackle structural inequalities and the roots of these inequalities in the migration regime. The existence of these activities and organizations indicate that these programmes alone are not sufficient to meet the existential needs of all subjects concerned. Moreover, these services, whether provided by the NGOs or the municipality, remain inside the scope of what Leerkes (2016) has described as “poor house policies”.

### 5.2. Alliance-building with other municipalities

The degree of cooperation between the local or regional governments plays an essential role regarding politicizing the issue against the federal government. In the Netherlands, the joint action of municipalities enabled, historically and currently, their decoupling from the central government’s approach. When the Linkage Act was approved, 170 of all 500 local authorities including the 4 big cities (Rotterdam, Utrecht, Amsterdam and Den Haag) protested the regulation and decided to keep sheltering NRAS (PICUM 2004). While the mayor of Amsterdam has been a central figure in promoting the provision of BBB-facilities, the city acted in accordance with several other, especially the larger municipalities. Based on the decision of the ECSR, the mayor of Amsterdam and, following his example, the Association of Netherlands Municipalities (VNG), have come forward to publicly advocate for access to social support for irregular migrants (*De Volkskrant,* 28 August 2014; Schapendonk 2016, 90), thereby directly opposing the national government. Organizing through VNG reinforced the collective position and common voice (Spencer 2017, 11) of Dutch municipalities and, subsequently, strengthened Amsterdam’s negotiating power vis-á-vis the national government.

In Vienna and Stockholm, we did not observe such strong political pressure as in Amsterdam by mobilizing in alliance with other municipalities. Horizontal processes connecting multiple local governments (Filomeno 2017) take place in these countries only through institutionally set up procedures. SALAR, the association for municipalities, county councils and regions in Sweden, acted mainly upon its advisory function and, contrary to VNG in the Netherlands, did not evolve as a political actor (Interview 5, 9, 13; mail correspondence with Interviewee 2, 3 March 2018). Stockholm’s low efforts in this regard match its marginal willingness to politicize the restriction of national services for NRAS. Similarly, in Austria, the “Landes-Flüchtlingsreferentenkonferenz” is a coordinating council between the provinces including the federal interior ministry to develop a joint approach for coordination and harmonized practices regarding access to welfare services and their quality. While at first, different implementation practices existed between the provinces, this institutional setting helped to coordinate and harmonize the access to shelter-facilities for NRAS (Interview 21 and 22). On the other hand, some acute collaborative political effort can be observed with other provinces, when the responsibilities of the regional governments are attacked by the central state. For instance, Vienna developed a joint position against the change of the Basic Welfare Support Agreement together with the provinces of Upper Austria, Tyrol, Styria and Salzburg (*Kurier* 2 March 2017).

Summing up, these horizontal relations are to a different extent crucial for strengthening the local position, especially in a top-down vertical setting. Amsterdam’s involvement in alliance building with other local governments enabled the city to enact a strong standing against the national government, which eventually enabled decoupling. To a lesser extent, this was also the case in Vienna, however, the networking platforms were primarily used for technical cooperation between the regional governments. This may be attributable to the regions’ relatively stable position and competences in the multi-level governance, which does not require much networking to strengthen its political position. Stockholm, which did not politicize the issue and accepted a top–down approach, refrained from collectively organizing with other municipalities for a common position, accordingly.

## 6. Conclusion

This article focussed on the multi-level governance setting in three cities, Amsterdam, Stockholm and Vienna, regarding a contested policy issue: welfare service provision for NRAS. The analysis shows that a restrictive national policy setting can produce different local responses: counter-politics in Amsterdam, shadow politics in Vienna, and non-issue in Stockholm. We have argued that, while the cities’ formal competences matter, they do not mark the limits of each response. Instead, we expand the MLG literature’s focus on vertical relations through underlining the significance of relationships to other local state and non-state actors in a multi-level governance. The literature on sanctuary cities and urban citizenship generally succeeds in grasping the importance of the relation between civil society actors and municipalities but fails to systematically analyse the importance of national institutional and legal frameworks in which these relations are embedded. By bringing these aspects together, we advocate that, on an analytical level, vertical and horizontal relations should be discussed as relational, rather than as separate issues.

In our analysis, we looked at how horizontal relations shape municipalities’ responses towards NRAS’ access to welfare services within a given national MLG setting. Firstly, we investigated the activities of NGOs and their degree of incorporation with the cities, shaping the position of the municipalities. This is obvious in Amsterdam and Vienna, where close cooperation between the cities and NGOs strengthened the position of the municipalities as well as the NGOs’ service capacities and, hence, resulted in more comprehensive services. However, the role of the NGOs in both cities cannot be reduced to their role as service providers. Especially in Amsterdam, activities of the NGOs for social rights advocacy or campaigning were decisive for pushing the issue towards a political agenda of the city government. In the case of Stockholm, we propose the thesis that the civil society’s “backyard activities” in Stockholm cushioned the potentially problematic effects of the national policy change and, hence, shaped the local response indirectly. Second, we focussed on the role of alliance-building with other local or regional governments. Here we observed that an effective use of networks between local governments strengthens the capabilities of local governments.

In order to refine and expand these findings, the analysis will have to be applied systematically to more than just one city in each national context. This would be particularly interesting for the Netherlands and Sweden, as in both countries other cities are also affected by the presence of NRAS with service needs, and some actively engage in dealing with the issue. As we focussed in this paper on the governance structure of welfare provision for NRAS, a thorough examination of the actual quality of these provided services came somewhat short – that is, whether they go beyond more or less basic forms of immediate, short-term poverty relief. With this in mind, we suggest further research examine practices of cities following a more repressive stance against civil society actors who support irregular migrants and NRAS. Another research gap is regarding the role of the EU-institutions and the connections of the cities on the European level that shape the response of individual city-governments which should be addressed in further research.

## References

[CIT0001] Ambrosini, M., and P. Boccagni. 2015. “Urban Multiculturalism Beyond the ‘Backlash’: New Discourses and Different Practices in Immigrant Policies Across European Cities.” *Journal of Intercultural Studies* 36 (1): 35–53. doi: 10.1080/07256868.2014.990362

[CIT0002] Ataç, I. 2019. “Deserving Shelter: Conditional Access to Accommodation for Rejected Asylum Seekers in Austria, the Netherlands, and Sweden.” *Journal of Immigrant & Refugee Studies* 17 (1): 44–60. doi:10.1080/15562948.2018.1530401. 31080376 PMC6485263

[CIT0003] Ataç, I., and S. Rosenberger. 2019. “Social Policies as a Tool of Migration Control.” *Journal of Immigrant & Refugee Studies* 17 (1): 1–10. doi:10.1080/15562948.2018.1539802. PMC6485261 31080375

[CIT0004] Bauder, H., and D. A. Gonzalez. 2018. “Municipal Responses to ‘Illegality’: Urban Sanctuary Across National Contexts.” *Social Inclusion* 6 (1): 124–134. doi:10.17645/si.v6i1.1273.

[CIT0005] Campomori, F., and T. Caponio. 2017. “Immigrant Integration Policymaking in Italy: Regional Policies in a Multi-Level Governance Perspective.” *International Review of Administrative Sciences* 83 (2): 303–321. doi: 10.1177/0020852315611238

[CIT0006] Cuadra, C. B. 2015. “Encounters With Irregular Migrants in Social Work – ‘Collateral Damage’ and Reframing of Recognizability in Swedish Public Social Services.” *Journal of Immigrant & Refugee Studies* 13 (3): 302–320. doi: 10.1080/15562948.2015.1037518

[CIT0007] de Graauw, E. 2015. “Polyglot Bureaucracies: Nonprofit Advocacy to Create Inclusive City Governments.” *Journal of Immigrant & Refugee Studies* 13 (2): 156–178. doi: 10.1080/15562948.2015.1030809

[CIT0008] Delvino, N. 2017. *European Cities and Migrants with Irregular Status: Municipal Initiatives for the Inclusion of Irregular Migrants in the Provision of Services. Report for the ‘City Initiative on Migrants with Irregular Status in Europe’*. Oxford: COMPAS, University of Oxford.

[CIT0009] EMN. 2016. *Returning Rejected Asylum Seekers: Challenges and Good Practices*. Common Template of EMN Focussed Study 2016.

[CIT0010] European Committee of Social Rights. 2014. CEC v. The Netherlands, Complaint No. 90/2013.

[CIT0011] Filomeno, F. A. 2017. *Theories of Local Immigration Policy*. London: Palgrave Macmillan.

[CIT0012] Guiraudon, V. 2004. “Immigration Reform in Comparative Perspectives: Sunshine and Shadow Politics in the United States and Europe.” In *Transatlantic Policymaking in an Age of Austerity: Diversity and Drift*, edited by M. Levin and M. Shapiro, 131–157. Washington, DC: Georgetown University Press.

[CIT0013] Hepburn, E., and R. Zapata-Barrero. 2014. *The Politics of Immigration in Multi-Level States: Governance and Political Parties*. London: Palgrave Macmillan.

[CIT0014] Kos, S., M. Maussen, and J. Doomernik. 2015. “Policies of Exclusion and Practices of Inclusion: How Municipal Governments Negotiate Asylum Policies in the Netherlands.” *Territory, Politics, Governance* 4 (3): 1–21.

[CIT0015] Leerkes, A. 2016. “Back to the Poorhouse? Social Protection and Social Control of Unauthorised Immigrants in the Shadow of the Welfare State.” *Journal of European Social Policy* 26 (2): 140–154. doi:10.1177/0958928716637139.

[CIT0016] Lippert, R. K., and S. Rehaag, eds. 2013. *Sanctuary Practices in International Perspectives: Migration, Citizenship and Social Movements*. Abingdon: Routledge.

[CIT0017] Lundberg, A., and M. Spång. 2017. “Deportability Status as Basis for Human Rights Claims: Irregularised Migrants’ Right to Health Care in Sweden.” *Nordic Journal of Human Rights* 35 (1): 35–54. doi: 10.1080/18918131.2017.1285953

[CIT0018] Lutz, F. 2018. “Non-removable Returnees Under Union Law: Status Quo and Possible Developments.” *European Journal of Migration and Law* 20 (1): 28–52. doi: 10.1163/15718166-12340019

[CIT0019] Nicholls, W. J. 2006. “Associationalism from Above: Explaining Failure Through France’s Politique de la Ville.” *Urban Studies* 43 (10): 1779–1802. doi: 10.1080/00420980600838135

[CIT0020] Oomen, B., and M. Baumgärtel. 2018. “Frontier Cities: The Rise of Local Authorities as an Opportunity for International Human Rights Law.” *European Journal of International Law* 29 (1): 607–630. doi: 10.1093/ejil/chy021

[CIT0001a] PICUM. 2014. *Housing and Homelessness of Undocumented Migrants in Europe: Developing Strategies and Good Practices to Ensure Access to Housing and Shelter*. Report, Brussels: PICUM.

[CIT0021] Rosenberger, S., and S. Koppes. 2018. “Claiming Control: Cooperation with Return as a Condition for Social Benefits in Austria and the Netherlands.” *Comparative Migration Studies* 6: 1. doi:10.1186/s40878-018-0085-3. 30221144 PMC6132690

[CIT0022] Schapendonk, M. 2016. *Reorganizing the provision of social support to homeless aliens in the Netherlands: A Bourdieusian perspective on the meaning of aliens’ welfare rights*. Radboud University Nijmegen: Master Thesis.

[CIT0023] Schiller, M. 2015. “Paradigmatic Pragmatism and the Politics of Diversity.” *Ethnic and Racial Studies* 38 (7): 1120–1136. doi:10.1080/01419870.2014.992925.

[CIT0024] Scholten, P. 2013. “Agenda Dynamics and the Multi-level Governance of Intractable Policy Controversies: the Case of Migrant Integration Policies in the Netherlands.” *Policy Sciences* 46 (3): 217–236. doi:10.1007/s11077-012-9170-x.

[CIT0025] Schoukens, P., and S. Buttiens. 2017. “Social Protection of Non-removable Rejected Asylum-Seekers in the EU: A Legal Assessment.” *European Journal of Social Security* 19 (4): 313–334. doi: 10.1177/1388262717744708

[CIT0026] Spencer, S. 2014. *The Sunshine and Shadow Politics of Irregular Migrants in Europe*. Oxford: COMPAS.

[CIT0027] Spencer, S. 2017. “Multi-level Governance of an Intractable Policy Problem: Migrants with Irregular Status in Europe.” *Journal of Ethnic and Migration Studies* 44 (12): 2034–2052. doi:10.1080/1369183X.2017.1341708.

[CIT0002a] Spencer, S., and N. Delvino. 2019. “Municipal Activism on Irregular Migrants: The Framing of Inclusive Approaches at the Local Level.” *Journal of Immigrant & Refugee Studies 17* (1): 27–43. doi:10.1080/15562948.2018.1519867.

[CIT0028] Squire, V., and J. Bagelman. 2012. “Taking not Waiting: Space, Temporality and Politics in the City of Sanctuary Movement.” In *Migration and Citizenship: Migrant Activism and the Politics of Movement*, edited by P. Nyers and K. Rygiel, 146–164. London, NY: Routledge.

[CIT0029] Swedish Red Cross. 2016. Lägesrapport 2016. Konsekvenser av ändringen i lagen om mottagande av asylsökande m.fl. (LMA), https://www.redcross.se/globalassets/press-och-opinion/rapporter/roda-korset_lagesrapport_-lma-2016.pdf.

[CIT0030] Triandafyllidou, A. 2010. “Irregular Migration in Europe in the Early 21st Century.” In *Irregular Migration in Europe. Myths and Realities*, edited by A. Triandafyllidou, 1–21. Aldershot: Ashgate.

[CIT0031] Utlåtande 2017:222 RVI (Dnr 150-2129/2016). Reviderade riktlinjer för handläggning av ekonomiskt bistånd. Förslag från socialnämnden. https://insynsverige.se/documentHandler.ashx?did=1906580.

[CIT0032] Van der Leun, J. 2003. *Looking for Loopholes: Processes of Incorporation of Illegal Immigrants in the Netherlands*. Amsterdam: Amsterdam University Press.

[CIT0033] Van Meeteren, M. 2014. *Irregular Migrants in Belgium and the Netherlands: Aspirations and Incorporation*. Amsterdam: Amsterdam University Press.

[CIT0003a] Van der Leun, J., and H. Bouter. 2015. “Gimme Shelter: Inclusion and Exclusion of Irregular Immigrants in Dutch Civil Society.” *Journal of Immigrant & Refugee Studies* 13 (2): 135–155. doi:10.1080/15562948.2015.1033507.

[CIT0034] List of interviews cited

[CIT0035] Interview 1. Head of facility and case worker at NGO in Stockholm, 23.01.2017.

[CIT0036] Interview 2. High level bureaucrat at a Swedish association of local authorities, 02.02.2017.

[CIT0037] Interview 3. High level bureaucrat at the Swedish national migration agency, 26.01.2017.

[CIT0038] Interview 4. High level bureaucrat at a Swedish association of local authorities, 22.03.2017.

[CIT0039] Interview 5. High level bureaucrat in the Stockholm social policy administration, 20.03.2017.

[CIT0040] Interview 6. Team leader at the Swedish Church, 03.02.2017.

[CIT0041] Interview 7. High level bureaucrat at the Swedish national migration agency, 21.03.2017.

[CIT0042] Interview 8. City lawyer in Malmö, 30.03.2017.

[CIT0043] Interview 9. Team leader and case worker at NGO in Stockholm, 01.02.2017.

[CIT0044] Interview 10. High level bureaucrat at the Swedish national migration agency, 21.03.2017.

[CIT0045] Interview 11. Chairperson at NGO in Stockholm, 31.01.2017.

[CIT0046] Interview 12. Project leader at NGO in Stockholm, 23.03.2017.

[CIT0047] Interview 13. High level bureaucrat at the Swedish national migration agency, 27.01.2017.

[CIT0048] Interview 14. Case worker at a NGO in Stockholm, 05.01.2018.

[CIT0049] Interview 15. Team leader and case worker at an NGO in Vienna, 24 October 2016.

[CIT0050] Interview 16. Legal and social counseler at an NGO in Vienna, 22 December 2016.

[CIT0051] Interview 17. Case worker at an NGO in Vienna, 10 November 2016.

[CIT0052] Interview 18. Team leader and case worker at an NGO in Vienna, 29 September 2016.

[CIT0053] Interview 19. Team leader and case worker at an NGO in Vienna, 27 September 2016.

[CIT0054] Interview 20. Head of legal counseling for asylum seekers at an NGO in Lower Austria, 21 November 2016.

[CIT0055] Interview 21. High level bureaucrat at Viennese Welfare Agency, 3 October 2016.

[CIT0056] Interview 22. Minister for integration and environment in the province of Upper Austria, 6 June 2017.

[CIT0057] Interview 23. Team leader at an NGO in Amsterdam which offers shelter, 23 March 2017.

[CIT0058] Interview 24. Member in the city council of Amsterdam, 20 April 2017.

[CIT0059] Interview 25. Program officer of a health-related NGO in Amsterdam, 14 April 2017.

[CIT0060] Interview 26. Policy officer migration, Municipality of Amsterdam, 29 Mar 2017.

[CIT0061] Interview 27. Social counsellor at an NGO in Amsterdam which offers shelter, 3 February 2017.

[CIT0062] Interview 28. Program officer of a health-related NGO in Amsterdam, 10 February 2017.

